# 支气管胸膜瘘—胸外科医生需要重视的肺部手术并发症

**DOI:** 10.3779/j.issn.1009-3419.2018.03.27

**Published:** 2018-03-20

**Authors:** 向阳 初

**Affiliations:** 解放军总医院胸外科 Department of Thoracic Surgery, PLA General Hospital

支气管胸膜瘘（bronchopulmonary fistula, BPF）是肺切除术后最严重的并发症之一，病死率高。随着外科技术进步、手术器械改进、围手术期处理的科学合理，BPF发病率已明显减少，总发生率在1%左右。Wright等^[1]^认为BPF应限制在全肺切除术后，尽量避免发生在肺叶切除术后。BPF常发生在术后7天-10天，也有少数发生在术后1个月以上的晚期BPF。BPF危险因素包括全身因素（如糖尿病）、长期应用糖皮质激素、低蛋白血症，术前接受新辅助放化疗，局部因素（支气管残端处理不佳、肿瘤残留、结核感染），手术能量器械使用不当也是引起BPF重要原因。BPF治疗有多种方法，对于病情危重或瘘口较小的BPF首选内镜治疗、封堵剂、支架置入等^[2]^；对于瘘口较大且全身情况较好可耐受再次手术者，可再次进胸直接缝合修补瘘口、或切除残端再次闭合瘘口，并用带蒂肌瓣、心包或大网膜加固残端^[3]^。大网膜血运丰富，抗炎再生能力强，是修补、加固瘘口的理想材料。进行带蒂大网膜移植时需要特别注意避免血管蒂的扭转，以确保大网膜血供不受影响，这是决定手术成功的关键。国内外多家单位采用带蒂大网膜治疗BPF取得较好的效果。Puskas等^[4]^应用大网膜修补25例BPF，成功23例；应用带蒂肌瓣修补14例BPF，成功9例，故认为大网膜移植封闭瘘口效果较好。高禹舜^[5]^应用大网膜加固 > 3 mm的瘘口残端，成功率83.3%（5/6）。成都医学院的杨晓樽医生应用大网膜胸腔内移植覆盖BPF修补后的残端，6例患者全部恢复良好，进一步证实了大网膜在有利于促进支气管残端的愈合，值得推广。大网膜移植手术应注意以下几点^[6]^：大网膜剪裁长度要充分，避免蒂扭转和过度牵拉；清除脓腔坏死组织要彻底；瘘口修补确切、不漏气；重视围手术期处理，纠正低蛋白血症、贫血及抗感染、抗结核。由于大网膜体积较小，脓腔较大不能充分填塞残腔时，可采用带蒂肌瓣（背阔肌、胸大肌、前距肌）封闭瘘口、填塞残腔。

随着医疗技术进步BPF的治疗效果较前大大提高，但是，对胸外科医师来说BPF仍然是巨大挑战。降低BPF发生率十分重要，以下几点需要特别注意^[7, 8]^：①清扫纵隔淋巴结时，尽量不要损伤支气管动脉，保护好支气残端周围的血供；②用合适厚度的缝钉闭合支气管残端，闭合后仔细检查残端是否有漏气，必要时加固缝合；③若支气管残端处理不满意，可用周围组织如心包，肋间肌，甚至大网膜包埋残端；④积极有效的处理胸腔积液和胸腔感染；⑤加强围手术期处理，如纠正贫血、低蛋白血症、减少激素用量；⑥使用能量器械清扫淋巴结时，注意避免损伤支气管残端和膜部。
